# A Rare Occurrence of Endometriosis Externa Individually Within the Rectus Abdominis Muscle

**DOI:** 10.7759/cureus.33662

**Published:** 2023-01-11

**Authors:** Ipek M Evruke, Andelib Babaturk, Gamze Akbas

**Affiliations:** 1 Obstetrics and Gynecology, Akcakoca State Hospital, Duzce, TUR; 2 Radiology, Akcakoca State Hospital, Duzce, TUR; 3 Pathology, Duzce Ataturk State Hospital, Duzce, TUR

**Keywords:** ultrasonography, abdominal wall, scar endometriosis, rectus abdominis muscle, endometriosis externa

## Abstract

Endometriosis is defined as the presence of ectopic functional endometrial tissue outside the uterine cavity. It most commonly occurs in the pelvic organs, including the ovaries, ligaments of the uterus, and the pouch of Douglas. Extrapelvic implantation of endometrial tissue has also been reported in the literature. Extrapelvic endometriosis can be explained by lymphatic/vascular migration or mechanical transplantation of the tissue during surgery. Rectus abdominis muscle endometriosis is a rare phenomenon that usually presents with a palpable abdominal mass and cyclic pain. Ultrasonography (USG), computed tomography (CT), and magnetic resonance imaging (MRI) can be effective to define the location and size of the mass. Our patient was a 32-year-old woman presenting with cyclic abdominal pain and the development of an abdominal mass she had noticed for a year. USG and MRI scans revealed an endometrial focus in the right rectus abdominis muscle. Surgical excision with negative margins was performed, since surgical treatment of the lesion is offered as the definitive treatment.

## Introduction

The classical definition of endometriosis is the presence of endometrial tissue outside of the uterine cavity. Endometriosis has an estimated prevalence of 10-15%, with a peak in women of reproductive age. Common implantation sites are pelvic organs, including the ovaries, ligaments of the uterus, and the pouch of Douglas. Extrapelvic endometriosis is relatively rare. Several theories have been reported for the implantation of extrapelvic endometriosis, including retrograde menstruation, mechanical transportation, coelomic metaplasia, and vascular/lymphatic metastasis.

The endometrial focus solely confined to the body of the rectus abdominis muscle is a very rare entity [1]. Here we present the case of a 32-year-old woman with 50×35×15 mm of endometriosis localized in the right rectus abdominis muscle treated with wide surgical excision.

## Case presentation

A 32-year-old woman presented with complaints of a palpable mass and cyclic pain in the lower right abdominal wall that she had noticed for a year. The pain was ongoing for a year, usually with exacerbations during the menses. The mass had overgrown and become even more palpable over the past four months, and also the pain had worsened and become non-intermittent with increased cyclic intensity.

She had a surgical history of three cesarean sections. The last cesarean section was three years ago. She had no concomitant medical conditions and no previous history of pelvic endometriosis. Clinical examination showed an irregular palpable and painful mass of 4-5 cm at the level of the lower right abdominal wall beneath the Pfannenstiel incision. Ultrasonography (USG) revealed a 42×43×17 mm heterogenous hypoechoic solid appearance in the right rectus abdominis muscle in the pubic region. Doppler ultrasonography revealed arterial blood supply; therefore, it supported the interpretation of the lesion as endometriosis.

**Figure 1:**
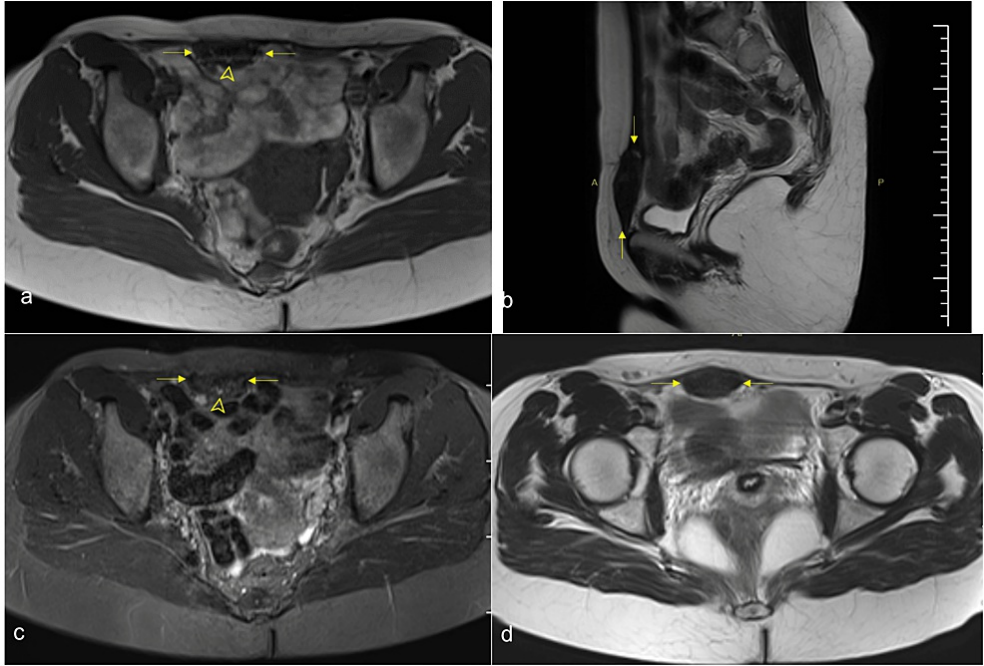
T1-weighted axial image (a), T2-weighted sagittal (b), and axial (d) images showed a hypointense lesion in the right rectus abdominis muscle (arrow). On the axial STIR sequence (c), which was at the same level of the image (a) a high internal punctate signal intensity became more conspicuous (arrowhead). STIR: short T1 inversion recovery.

A pelvic magnetic resonance imaging (MRI) was performed. MRI showed a relatively well-defined lesion of size 50×35×15 mm in the right rectus abdominis muscle. The lesion had a low signal intensity on both T1 and T2 weighted images suggestive of fibrosis. There was a high internal punctate signal intensity within the lesion seen on both T1 and T2-weighted images and became more significant on fat-suppressed images (Figure 1).

**Figure 2:**
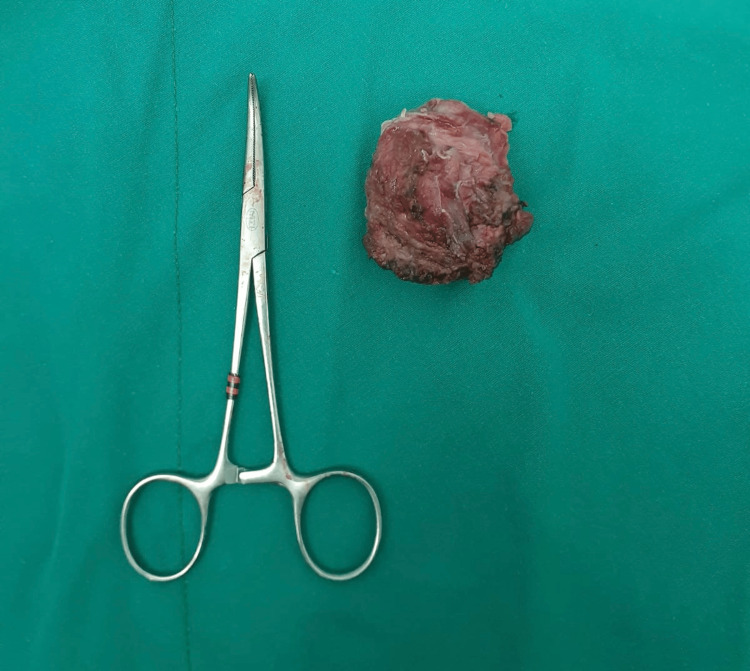
Postoperative specimen of the excised endometrial tissue from the rectus abdominis muscle.

Complete excision of the endometriotic focus in the right rectus abdominis muscle with 10 mm clear surgical resection margins was performed. No connection with intraabdominal structures was found. The aponeurosis defect was repaired tension-freely primarily using sutures no: 1 polyglactin (Figure 2).

**Figure 3:**
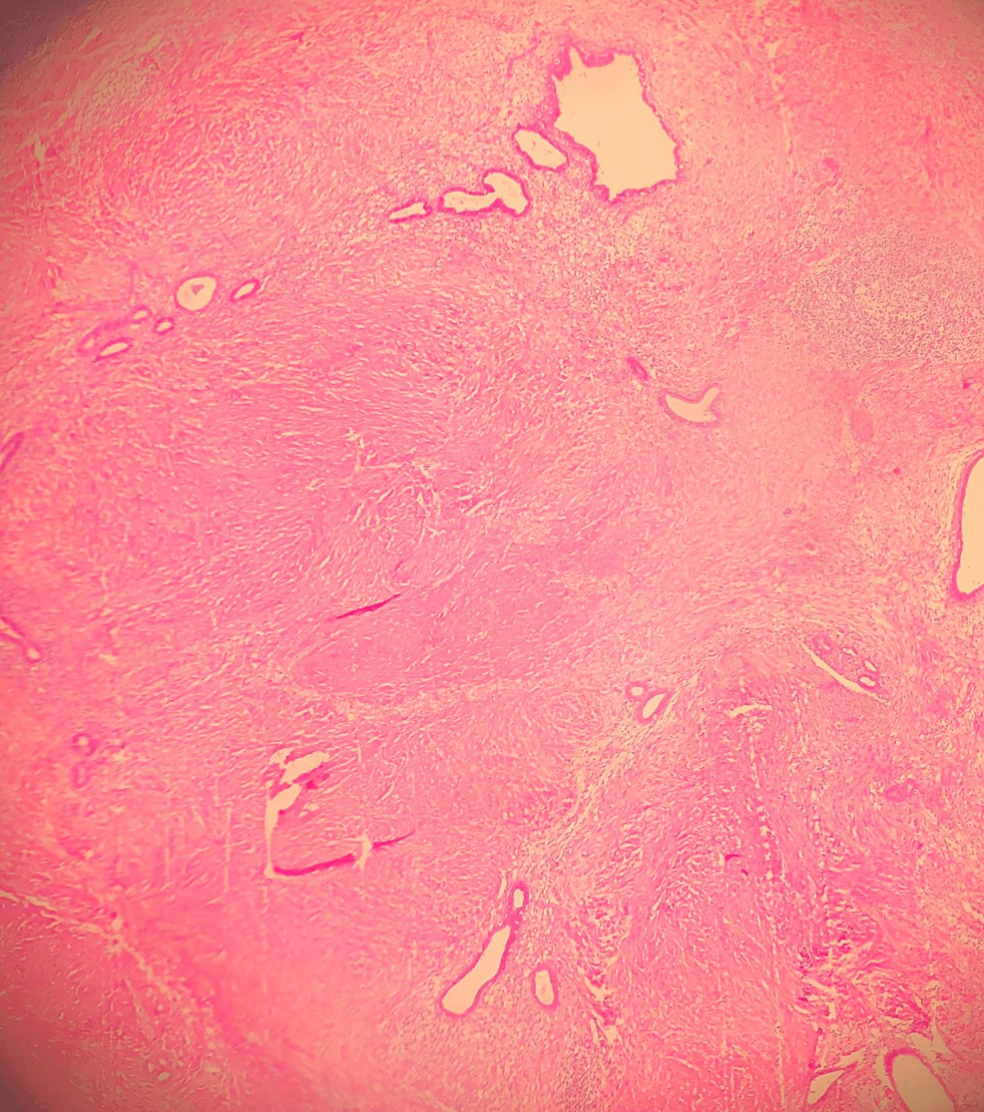
A histological aspect of endometrial gland structures and endometrial stroma included within the muscle fibers (H&E stain, 40× magnification).

**Figure 4:**
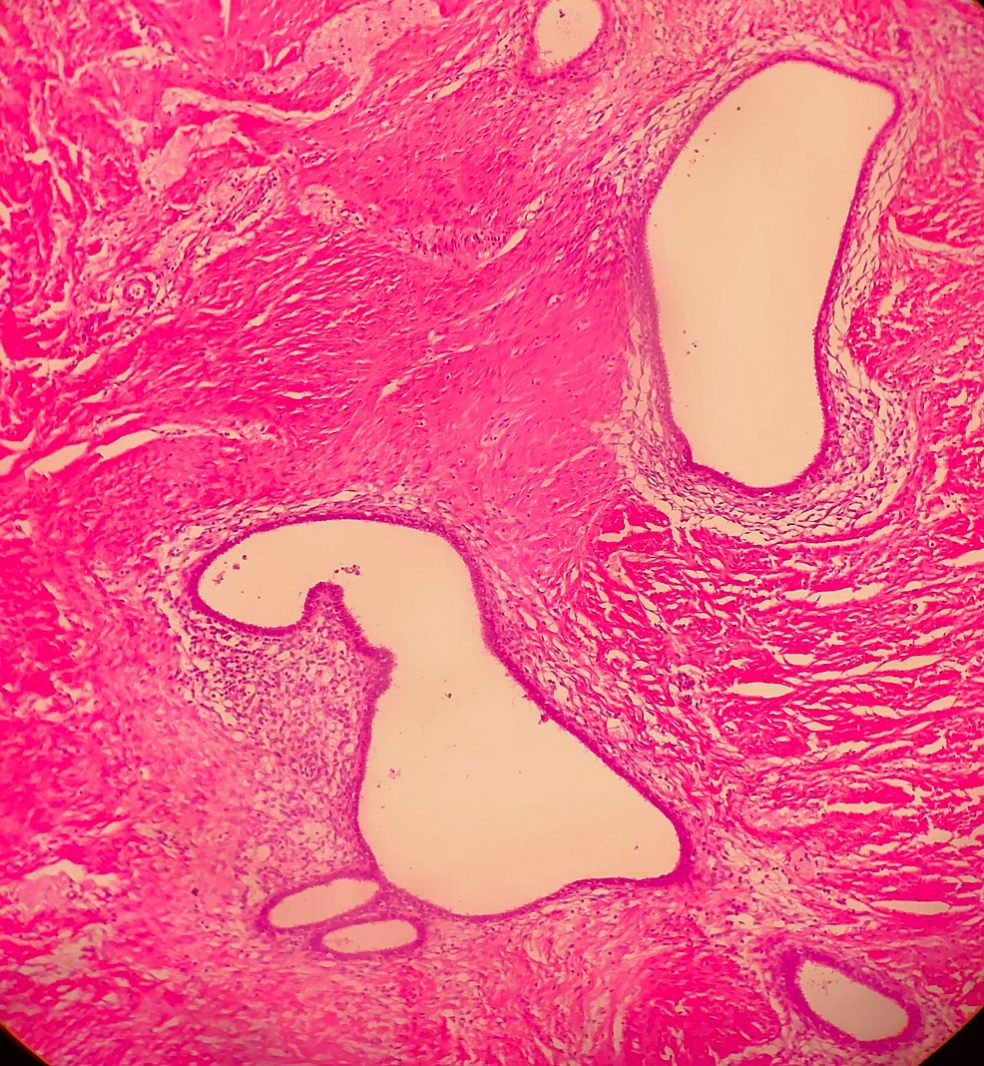
A histological aspect of endometrial gland structures and endometrial stroma included within the muscle fibers (H&E stain, 100× magnification).

**Figure 5:**
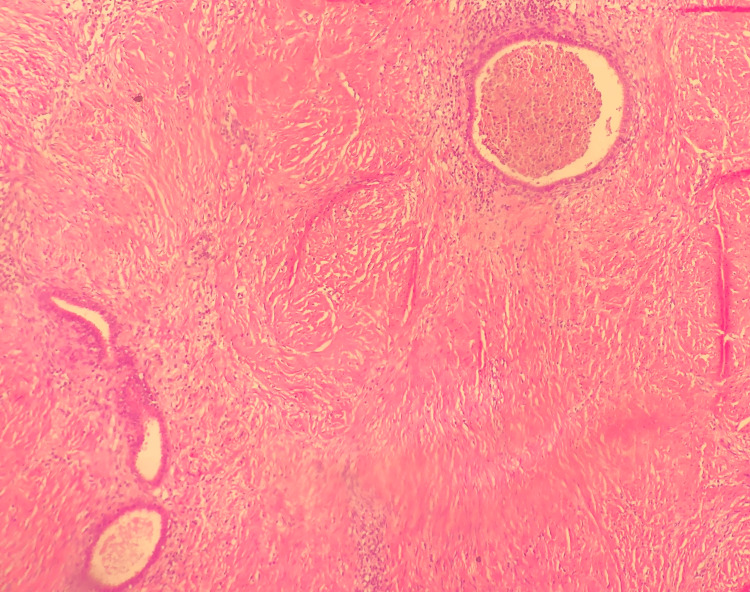
Ectopic endometrial tissue showing foci of hemorrhage with hemosiderin-laden histiocytes.

The histopathological report showed a 5×4×3 cm-sized, well-circumscribed burgundy-colored nodular fibrous tissue macroscopically. Inactive endometrial gland structures and endometrial stroma with hemosiderin-laden macrophages in the lumen of various sizes scattered randomly between muscle bundles were observed microscopically, consistent with the diagnosis of endometriosis of the rectus abdominis muscle (Figures 3-5). Postoperatively, the patient was discharged with relief of her symptoms. The patient has been in follow-up for 12 months with no sign of recurrence.

## Discussion

The endometrial gland and stroma that occur outside the uterine cavity are defined as endometriosis. It is usually located in the endopelvic organs and peritoneum, but it may be localized at extrapelvic locations such as the bowel, diaphragm, pleural cavity, and abdominal wall as well. It is characterized by chronic inflammatory reactions that cause dysmenorrhea, dyspareunia, chronic pain, and infertility. The peak age for the disease is between 25 and 45 years, with a prevalence of 10-15% in reproductive age, but it may also affect adolescents and menopausal women [2]. Endometriosis has a big impact on social and sexual life. Pain-related symptoms worsen the quality of life. Dyspareunia and infertility may harm partner relationships.

Endometriosis in the rectus abdominis muscle is a rare phenomenon of extrapelvic endometriosis and was first described in 1984 by Amato and Levitt [3]. There are multiple theories for extrapelvic endometriosis, including retrograde menstruation, lymphatic or vascular dissemination, and mechanical transplantation. Abdominal wall endometriosis is usually located in the subcutaneous tissue, with or without the extension to the fascia of the rectus abdominis. Our case is a rare form of abdominal wall endometriosis that is located individually within the rectus abdominis muscle. Endometriosis in the rectus abdominis muscle is usually iatrogenic and highly related to abdominal surgeries such as cesarean section, hysterectomy, appendectomy, and laparoscopic procedures [4].

Patient symptoms, the history of abdominal surgeries, and the physical examination play a big role in the diagnosis of abdominal wall endometriosis. The presence of a palpable mass on the abdomen and cyclic abdominal pain is highly suggestive of the diagnosis. The differential diagnosis of cyclic abdominal pain includes desmoid tumors, abscesses, incisional hernias, hematomas, granulomas, lymphadenitis, and even metastatic cancer.

Saliba et al. reported two cases of abdominal wall endometriosis. One of them was a 36-year-old woman presenting with cyclic abdominal pain and a history of cesarean surgery several years before the symptoms. The pain did not improve with hormonal therapy. They revealed a 3×3 cm mass at the lower rectus wall with physical examination and computed tomography (CT) scan that was treated by surgical excision. The second case was a 40-year-old woman presenting with cyclic abdominal pain and a history of a previous cesarean section. They revealed endometriosis focus in the rectus abdominis muscle by USG and CT and treated it with surgical excision. Both cases had no recurrence of the symptoms afterward at their follow-ups [5].

Giannella et al. reported a case of a 32-year-old woman presenting with cyclic pain. She had no previous surgery history or known pelvic endometriosis. An MRI scan confirmed a 2 cm mass individually in the left rectus abdominis muscle with no involvement of the intrabdominal cavity. A wide surgical excision was performed, and the patient showed no sign of recurrence during her four-year follow-up. Giannella et al. also reviewed 18 reported cases of rectus muscle endometriosis, which was prevalent in 27-42 years of age women. A case of 7×7 cm mass was reported by Kocakusak et al. as the largest size in this review [6,7].

Menon et al. reported a case of a 31-year-old woman presenting with lower abdominal pain. She had two previous cesarean sections. They revealed a 6×3.3 cm heterogenous mass present in the lower rectus abdominis muscle extending to the bladder and anterior of the uterus with physical examination and CT scan. They performed a total hysterectomy with wide surgical excision of the endometriotic focus. The abdominal wall defect was repaired with polypropylene mash due to the size of the endometriotic focus [8]. Khan et al. conducted a study that included 2539 women who had surgery for endometriosis. Of these, 34 women (1.34%) had abdominal wall endometriosis with 41% of the cases diagnosed clinically. Among these cases, 59% had endometriosis at the cesarean section scar [9].

Davis et al. presented a case of abdominal wall endometriosis with a literature review of 39 cases between 1999 and 2020. The most common symptom was an abdominal mass with cyclic pain presented in 31 of the patients (31/39, 79.5%). Thirty-seven of them had at least one previous pelvic surgery (37/39, 94.9%). About 100% of the patients in the review had undergone wide surgical excision [10]. USG followed by CT/MRI scanning is accepted as the definitive imaging in the diagnosis. Medical treatment for extrapelvic endometriosis is not offered as the initial therapy. Medical therapy such as oral contraceptives, progestogens and hormone suppression therapy with gonadotropin-releasing hormone (GnRH) may be used for patients who are unable to undergo surgery or as an adjuvant therapy postoperatively to prevent recurrence [7]. Wide surgical excision with negative margins is accepted as the definitive management of abdominal wall endometriosis.

## Conclusions

We presented a rare form of extrapelvic endometriosis isolated in the rectus abdominis muscle. The time of the appearance of symptomatic endometriosis focus in the rectus abdominis varies, therefore making it a challenge in diagnosis. USG is a first-line tool in diagnostics. Transvaginal ultrasound is also helpful in examining pelvic structures since it is estimated that 25% of women with extrapelvic endometriosis coexist with pelvic endometriosis. CT and MRI also provide valuable information in diagnostics. Surgical excision is known to be the best definitive treatment for this exceptional condition. Surgical material should include at least 10 mm of clear surgical resection margins in order to minimize the risk of recurrence. Abdominal wall endometriosis must be kept in mind as one of the differential diagnoses in reproductive women presenting with abdominal pain due to the significant increase in cesarean sections.
